# Picosecond optical vortex pulse illumination forms a monocrystalline silicon needle

**DOI:** 10.1038/srep21738

**Published:** 2016-02-24

**Authors:** Fuyuto Takahashi, Katsuhiko Miyamoto, Hirofumi Hidai, Keisaku Yamane, Ryuji Morita, Takashige Omatsu

**Affiliations:** 1 Graduate School of Advanced Integration Science, Chiba University, 1-33 Yayoi-cho, Inage-ku, Chiba 263-8522, Japan; 2 Molecular Chirality Research Center, Chiba University, 1-33 Yayoi-cho, Inage-ku, Chiba 263-8522, Japan; 3 Graduate School of Mechanical Engineering, Chiba University, 1-33, Yayoi-cho, Inage-ku, Chiba, 263-8522, Japan; 4 Department of Applied Physics, Hokkaido University, Kita-13, Nishi-8, Kita-ku, Sapporo, Hokkaido 060-8628, Japan

## Abstract

The formation of a monocrystalline silicon needle by picosecond optical vortex pulse illumination was demonstrated for the first time in this study. The dynamics of this silicon needle formation was further revealed by employing an ultrahigh-speed camera. The melted silicon was collected through picosecond pulse deposition to the dark core of the optical vortex, forming the silicon needle on a submicrosecond time scale. The needle was composed of monocrystalline silicon with the same lattice index (100) as that of the silicon substrate, and had a height of approximately 14 μm and a thickness of approximately 3 μm. Overlaid vortex pulses allowed the needle to be shaped with a height of approximately 40 μm without any changes to the crystalline properties. Such a monocrystalline silicon needle can be applied to devices in many fields, such as core–shell structures for silicon photonics and photovoltaic devices as well as nano- or microelectromechanical systems.

Optical vortices^1^,^2^,^3^, carrying an annular intensity profile and an orbital angular momentum arising from a helical wavefront, have provided a variety of research opportunities, such as optical manipulation^4^,^5^, super-resolution microscopes that function beyond the diffraction limit^6^,^7^, space-division multiplexing optical telecommunications^8^,^9^, and quantum information^10^,^11^. In particular, we have proposed a method for materials processing in which optical vortices force a metal to form structured materials, including nanoneedles^12^,^13^ and chiral structures^14^,^15^,^16^, owing to orbital angular momentum transfer effects.

This technique of forming structured metallic materials by the optical vortex illumination of a metal target will allow us to improve the time and cost efficiencies of fabricating advanced plasmonic devices.

Monocrystalline silicon has been widely investigated as a base material in a variety of photonic devices, such as photonic crystals^17^,^18^, optical waveguides^19^,^20^, photovoltaic devices^21^,^22^, field emission arrays^23^,^24^, and metamaterials in a terahertz range^25^,^26^. In particular, monocrystalline silicon nano- or microstructures^27^,^28^,^29^,^30^,^31^,^32^,^33^ instead of polycrystalline or amorphous silicon ones exhibit low optical scattering losses. They also enable significant enhancements in the performance of the above mentioned photonic devices. Optical vortex illumination will enable us to provide highly cost- and time-efficient fabrications of silicon nanostructures.

In this paper, we report on the first demonstration concerning the fabrication of a monocrystalline silicon needle with a nanoscale tip by illumination with a picosecond optical vortex pulse (single optical vortex pulse deposition). We also investigated the temporal evolution of the formation of a silicon needle, and revealed for the first time how the silicon needle is formed by optical vortex pulse illumination. After the deposition of a picosecond vortex pulse, the silicon needle was recrystallized to be monocrystalline on a submicrosecond time scale. Several overlaid vortex pulses shaped the needle with a height of approximately 40 μm.

## Results

**Figure 1:**
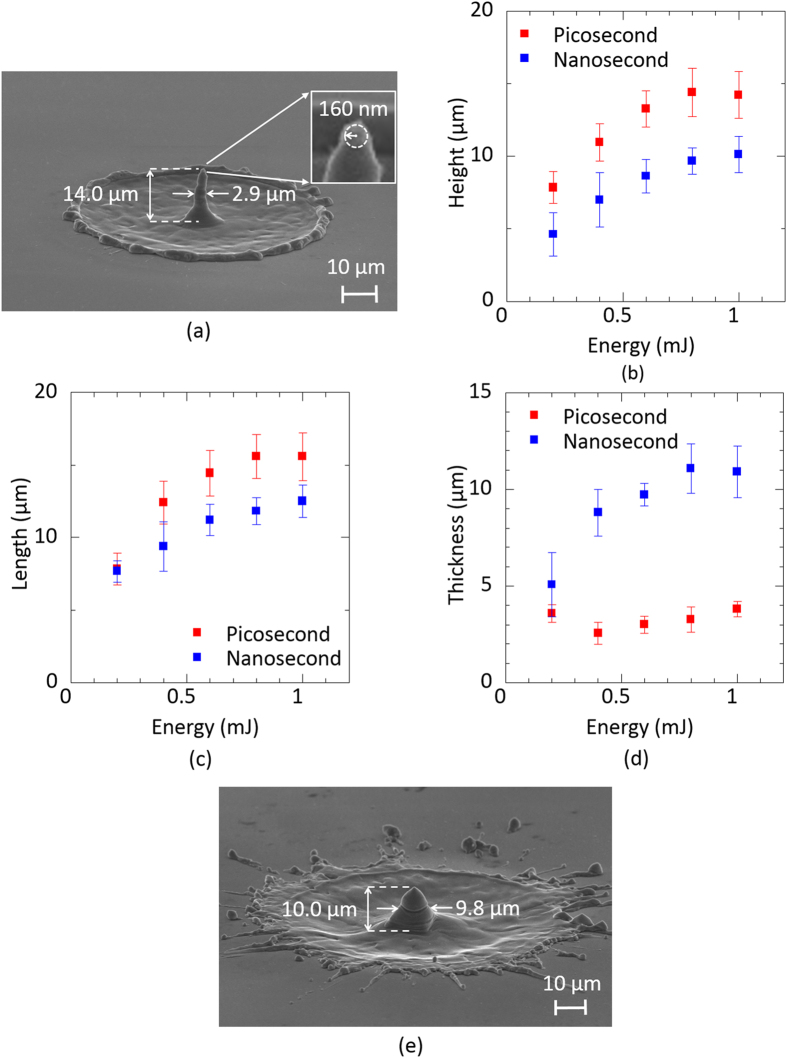
(**a**) Silicon needle fabricated by irradiation of a picosecond vortex pulse with an energy of 0.6 mJ. The inset shows a magnified silicon needle tip. (**b**) Experimental height of the needles versus the vortex pulse energy. Experimental data plots by the illuminations of picosecond and nanosecond vortex pulses are shown by red and blue colors. Error bars mean the standard deviations of the measured values. (**c**) Experimental length of the needles versus the vortex pulse energy. Experimental data plots by illuminations of picosecond and nanosecond vortex pulses are shown by red and blue colors. Error bars mean the standard deviations of the measured values. (**d**) Experimental thickness of the needles versus the vortex pulse energy. Experimental data plots by illuminations of picosecond and nanosecond vortex pulses are shown by red and blue colors. Error bars mean the standard deviations of the measured values. (**e**) Silicon needle fabricated by the irradiation of a nanosecond vortex pulse with an energy of 0.6 mJ.

Illumination by a picosecond optical vortex pulse (single vortex pulse deposition) with an energy of 0.6 mJ, which is sufficiently higher than the ablation threshold of approximately 0.03 mJ determined by using the crater method^34^, was used in this study to fabricate a needle-shaped structure (termed ‘silicon needle’) with little debris, as shown in Fig. 1(a). The optical vortex pulse was right-handed and carried a topological charge *l* of +1 and a spin angular momentum *s*, which is associated with a circular polarization, of +1.

The height of the needle, which is defined as the length between the silicon surface and the top of the needle, increased with increasing the picosecond pulse energy, and reached approximately 14 μm at a pulse energy of 0.6 mJ. (The ‘length’ of the needle, defined as the length between the top and bottom ends of the needle was also measured to be 15 μm.) The height and length of the needle were measured by utilizing a laser scanning microscope (Keyence VK-9700/VK9710GS; depth resolution, 14 nm). Also, the tip curvature radius of the needle, which was measured by fitting the tip with a circle, was then measured to be 160 nm. In contrast, the thickness, defined as the full-width at 50% height of the needle, remained unchanged at approximately 2.9 μm with increasing pump energy. Figure 1(b–d) show the experimental height, length and thickness of the needle as a function of the pulse energy.

Worse heating effects arising from high energy pulse illumination will generally suppress or erase the formation of nanostructures. In fact, nanosecond pulse illumination (wavelength, 1064 nm; pulse energy, 0.6 mJ; pulse duration, 20 ns) provided only a bump-shaped structure with debris arising from significant heat diffusion effects (Fig. 1(e)). The height and length of the fabricated bump were also limited to be ~10 μm and ~12 μm, even during the high energy pumping. Further, the measured thickness of approximately 9.8 μm was >3 times that of the silicon needle obtained by picosecond pulse illumination (Fig. 1(b–d)). These results indicate that picosecond pulse illumination allows for the suppression of undesired debris owing to heat diffusion effects and enables the formation of structured materials (sharp silicon needles).

**Figure 2:**
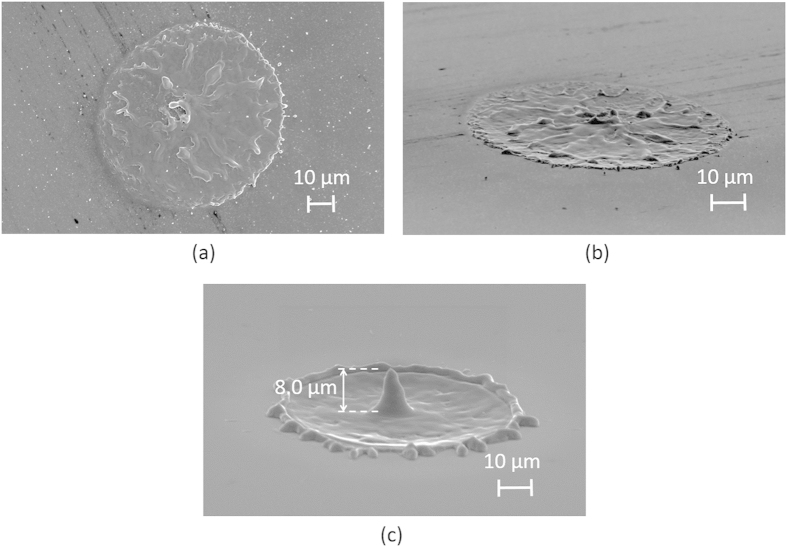
(**a**) Silicon surface ablated by a femtosecond vortex pulse with an energy of 0.2 mJ. (Top view) (**b**) Silicon surface ablated by a femtosecond vortex pulse with an energy of 0.2 mJ. (Side view) (**c**) Silicon needle structured by a picosecond vortex pulse with an energy of 0.2 mJ.

Surface structuring by the illumination of 20~100 overlaid femtosecond vortex pulses, in which ripples (or grooves) are formed along a radial or azimuthal (or spiral) polarization direction, has also been demonstrated^35^,^36^. The single femtosecond vortex pulse deposition (wavelength, ~800 nm; pulse energy, ~0.2 mJ; pulse width, ~200 fs) used in our study ablated the silicon surface without thermal melting, thereby creating only an annular crater without any ripples arising from the interaction between the incident and the surface-scattered lights (Fig. 2(a,b)). In contrast, the single picosecond pulse (pulse energy, 0.2 mJ) deposition still structured a needle with a height of 8.0 μm (Fig. 2(c)). Such needle formation requires the optical vortex pulse with a pulse duration of at least 10 ps so as to create the thermally-melted silicon.

**Figure 3:**
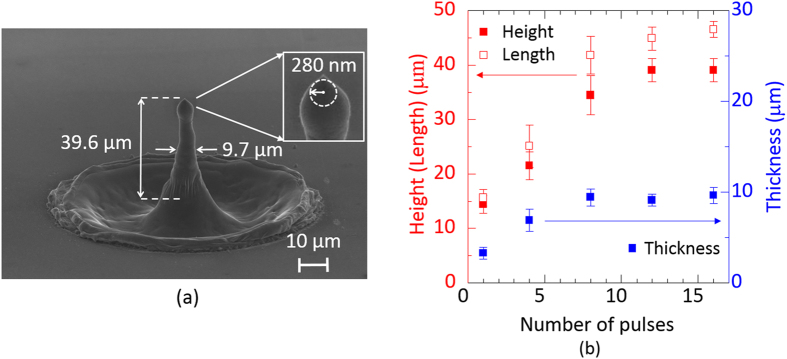
(**a**) Silicon needle fabricated by the irradiation of 12 overlaid vortex pulses. The inset shows a magnified silicon needle tip. (**b**) Experimental height (red square), length (open square) and thickness (blue square) of the needles as a function of the number of overlaid vortex pulses. The error bars mean the standard deviations of the measured values.

Several overlaid picosecond vortex pulses further caused the height of the needle to increase, as shown in Fig. 3(a). The pulse energy was then fixed at 0.8 mJ. When 12 picosecond vortex pulses were superimposed on the surface, the height (length) of the needle reached approximately 40 μm (47 μm) with a thickness of approximately 9.7 μm (a tip curvature of 280 nm). The experimental height and thickness of the needle are plotted against the number of overlaid picosecond vortex pulses in Fig. 3(b).

**Figure 4:**
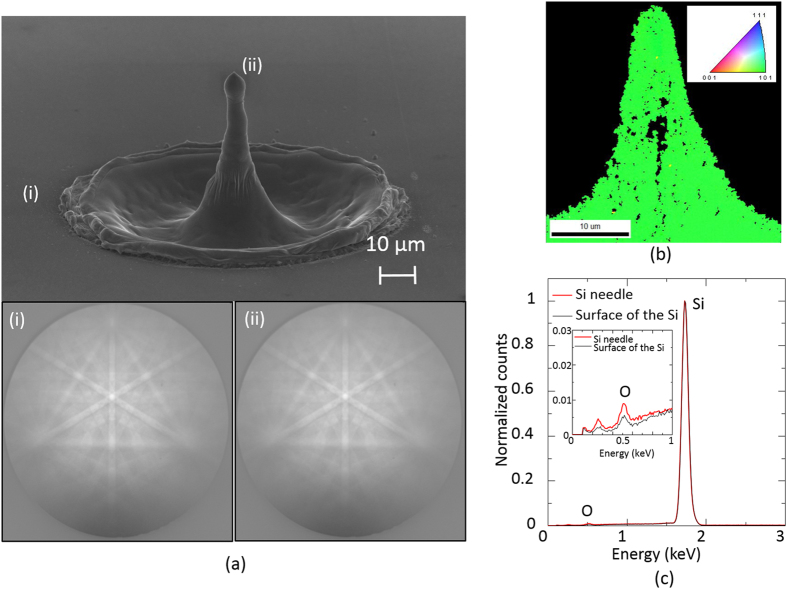
(**a**) Electron diffraction patterns of (i) the monocrystalline silicon substrate and (ii) the fabricated silicon needle. Twelve vortex pulses were then overlaid. (**b**) Spatial distribution of the lattice index in the fabricated silicon needle. The cross section of the needle exhibits a lattice index (101). Twelve vortex pulses were then overlaid. (**c**) Energy-dispersive X-ray (EDX) spectra of the silicon needle and the silicon substrate.

The lattice index of the needle fabricated by overlaid picosecond vortex pulse irradiation was investigated by an electron backscattering diffraction analysis. As shown in Fig. 4(a,b), the diffraction pattern of the silicon needle was identical to that of the silicon substrate with a lattice index of (100), although submicron-sized voids due to thermal shock by illumination with optical vortex pulses were seen inside the needle. These results indicate that the fabricated needle was recrystallized to be monocrystalline on the silicon substrate. Energy-dispersive X-ray (EDX) spectrum of the silicon needle covered with a thin SiO_2_ layer with a nanometer-scale thickness was almost identical to that of the silicon substrate (Fig. 4(c)).

## Discussion

Recrystallization of the silicon by ultrafast (femtosecond or picosecond) pulse laser irradiation was investigated in previous studies^37^,^38^, which focused mostly on the formation of polycrystalline silicon materials. Such monocrystalline silicon formation generally requires a low removal rate for the latent heat stored in the melted (liquid-phase) silicon.

**Figure 5:**
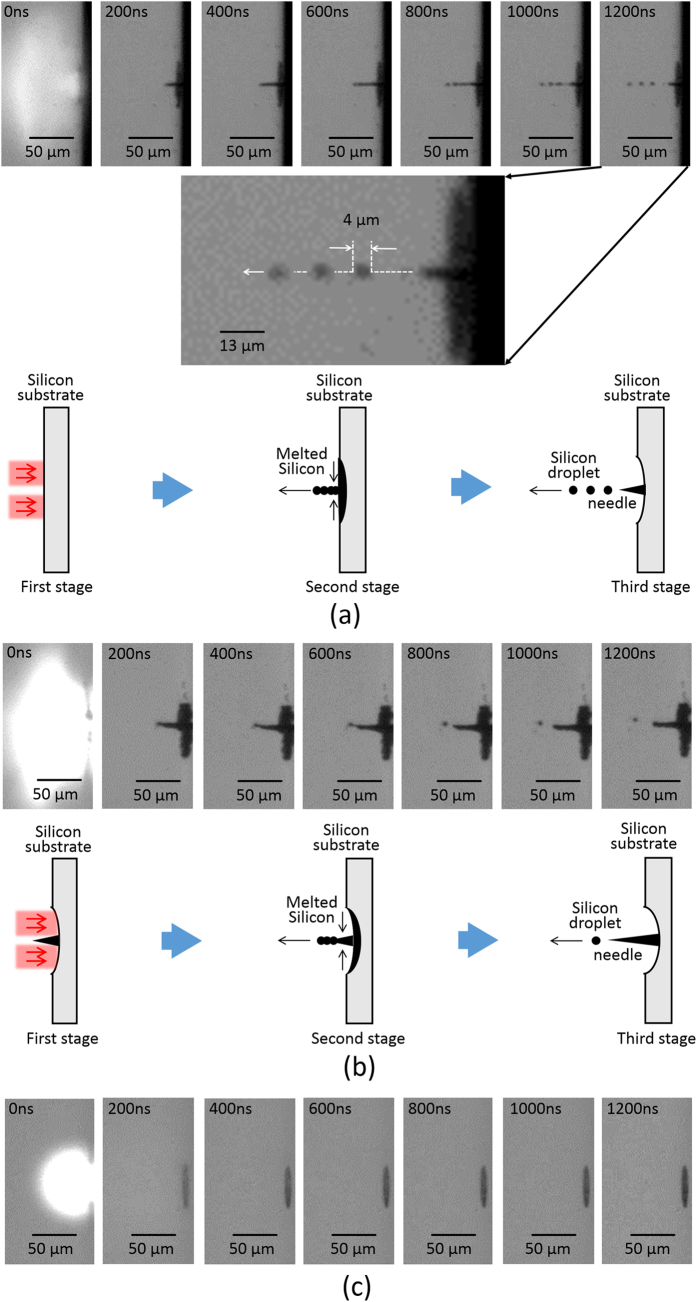
(**a**) Temporal evolution of silicon needle formation visualized by employing an ultrahigh speed camera during the first vortex pulse irradiation. The temporal evolution is classified under three stages. (First stage) The silicon is melted by the optical vortex pulse deposition. (Second stage) After the vortex pulse is gone, the melted silicon is collected to the dark core of the optical vortex. (Third stage) The silicon is accumulated at the core. Also, the silicon droplets then fly away. The resulting silicon needle is established. The inset shows a magnified silicon droplets. They fly to form a straight line, and their diameter (radius) is typically estimated to be ~4 (~2) μm. (**b**) Temporal evolution of the silicon needle formation visualized by employing the ultrahigh speed camera during a twelfth vortex pulse irradiation onto the silicon target. The temporal evolution is classified under three stages. (First stage) The silicon is melted by the optical vortex pulse deposition. (Second stage) After the vortex pulse is gone, the melted silicon is accumulated onto the silicon needle. (Third stage) The undesired silicon droplets then fly away. The height of the silicon needle is reinforced. (**c**) Temporal evolution of the ablated silicon surface visualized by employing the ultrahigh speed camera upon the irradiation of a picosecond pulse with a Gaussian spatial form. The thermal explosion and melting of the silicon occurred within 200 ns, thus resulting in shallow crater formation without any needles.

The temporal evolution of the silicon needle formation was investigated by employing an ultrahigh speed camera, as shown in Fig. 5(a).

The silicon is melted by optical vortex pulse deposition, and then receives optical radiation forces, such as an optical angular momentum and a forward scattering force, provided by the optical vortex pulse. The scattering force *F*_*s*_(*r*)^39^ produced by the optical vortex is


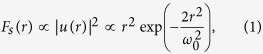


where |*u*(*r*)|^2^ is the optical intensity profile of the optical vortex, *r* is the radial coordinate of the optical field, and *ω*_0_ is the beam waist of the vortex pulse on the Si substrate. The resulting radial gradient ∆*F*(*r*) of the scattering force near the dark core (low-intensity region, *r* < *ω*_0_) is then established to be


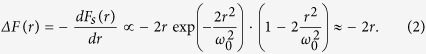


The radial gradient ∆*F*(*r*) will play the role of a restoring force, thereby collecting the melted silicon within the dark core of the optical vortex. In a similar manner, the vaporization-induced recoil pressure also ejects the melted silicon to the dark core or the outer region (first stage).

After approximately 200–600 ns, *i.e.*, after the vortex pulse is gone (the recoil pressure is also gone), the ejected silicon in the outer region is super-cooled, and it then expands with the recrystallization (because the solid-phase silicon has a lower mass density *ρ*_0_ (2.3 g/cm^3^)^40^ than that *ρ*_1_ (2.6 g/cm^3^)^40^ of the liquid silicon). Further, the silicon near the dark core is melted and shrunken by thermal diffusion effects. Such solid-liquid hydrodynamics with a submicrosecond time scale also acts as a mass transport driving force to collect the melted silicon within the dark core of the optical vortex (second stage).

An additional 200–600 ns later, the silicon is accumulated at the core. When the pulse energy is sufficiently higher than the ablation threshold, the optical vortex pulse also creates superfluous silicon droplets with a submicron size owing to a capillary-wave instability. The radius *a* of the droplets formed in the capillary-wave instability is estimated to be 1.3 μm, by using the following formula^41^:


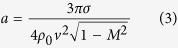


where σ is the surface tension coefficient (770 mN/m), *v* is the velocity of the silicon droplet (~50 m/s; the velocity was estimated from Fig. 5(a)) and *M* is the Mach number (~0.3), respectively. The estimated droplet radius is very consistent with experimental one (~2.0 μm) shown in Fig. 5(a). The instability time constant 1/*Γ*^40^defined as


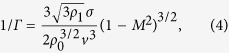


was also estimated to be ~7 ns. Thus, the required time for ejecting the silicon droplets is expected to be at least >10 ns.

Also, the silicon droplets then flew away to form a straight line, thus establishing a silicon needle with a nonspiral conical surface (third stage).

Such slow motion of the melted silicon (at a microsecond time scale) resulted in the recrystallization of the monocrystalline silicon needle. Further, the deposition of an overlaid vortex pulse increased the height of the silicon needle at a submicrosecond time scale, as shown in Fig. 5(b).

The ultrafast laser-induced explosion and melting might enable us to structure a nanoneedle (nanojet) on a target surface^42^. A circularly polarized pulse deposition with a Gaussian spatial form was irradiated on the silicon. The thermal explosion and melting of the silicon then occurred rapidly (<200 ns), thus resulting in a shallow crater formation without any needles (see Fig. 5(c)). These results evidence that the structured optical radiation forces based on optical vortex pulses induce the slow temporal dynamics of the melted silicon so as to form the monocrystalline silicon needle obtained in our study.

**Figure 6:**
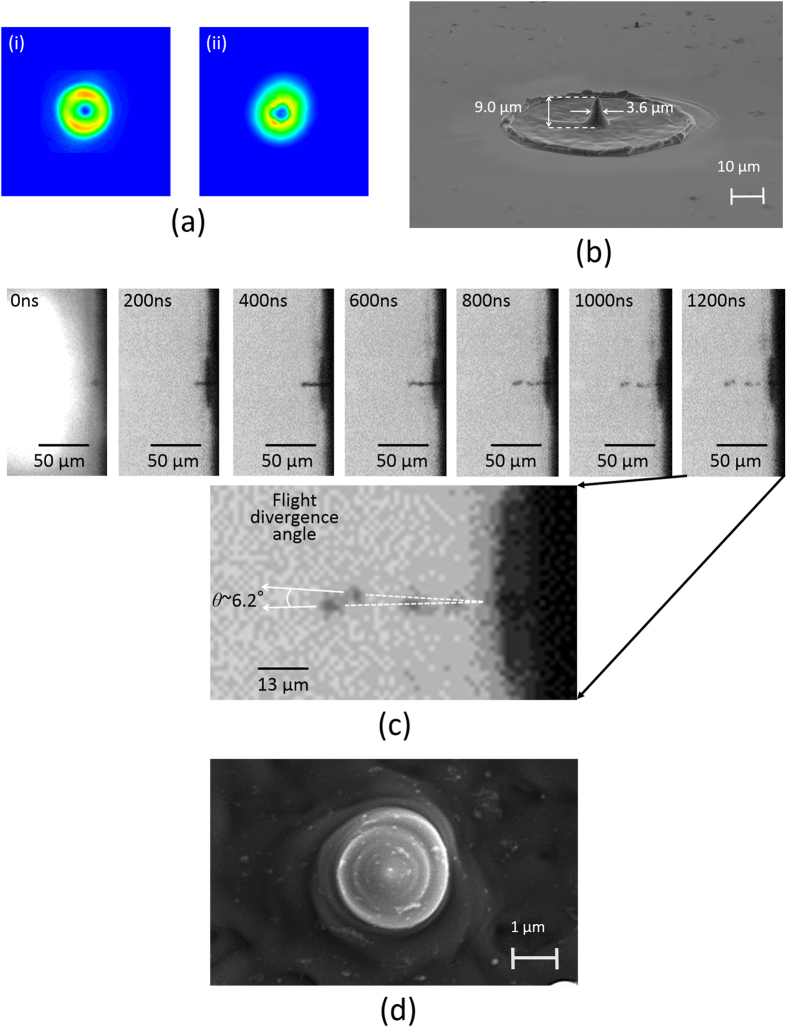
(**a**) Intensity profiles of (i) focused vortex and (ii) annular beams. (**b**) Silicon cone fabricated by illumination of a circularly polarized annular beam. (**c**) Temporal evolution of the silicon cone formation visualized by employing the ultrahigh speed camera upon annular beam irradiation onto the silicon target. The inset shows magnified silicon droplets with a wide divergence angle *θ* (>6^o^) of flight. (**d**) Right-handed chiral silicon structure fabricated by a low energy (near ablation threshold) vortex pulse pumping.

To clarify how the orbital angular momentum contributes to silicon needle formation, a circularly polarized annular beam without any orbital angular momentum, produced by a damaged folding mirror (the focused spatial form of the annular beam is shown in Fig. 6(a)), was also irradiated on the silicon. The pulse energy was then fixed at 0.8 mJ. The fabricated silicon structure was shaped to be a cone and its height and thickness were measured to be ~9.0 μm and ~3.6 μm, respectively (Fig. 6(b)).

In fact, the silicon droplets ejected from the silicon substrate by illumination of the annular beam pulse showed a wide divergence angle *θ* of flight (>6^o^), so as to impact efficient accumulation of the silicon on the substrate (Fig. 6(c)).

These results suggest that optical vortex pumping should provide a spin on the silicon droplets by total angular momentum transfer effects, resulting in that the silicon droplets fly so as to form a straight line, and are efficiently accumulated in the dark core to form the needle on the substrate. In fact, the low-energy (0.05 mJ) picosecond pulse deposition forced the melted silicon to form a chiral structure (Fig. 6(d)), evidencing that the melted silicon was spinning by orbital angular momentum transfer arising from the picosecond optical vortex pulse illumination.

At this moment, any direct observation of the spinning motion of the droplets was mainly impacted by the spatial resolution (~1.2 μm) of the high-speed camera. The spinning motion of the droplets should be further investigated, for instance, by utilizing nanosecond pump–probe analysis with high spatial and temporal resolutions.

As shown in our previous publication^15^, the spin angular momentum may constructively or destructively couple with the orbital angular momentum to shape the structures.

In our experiments based on loosely focused optical vortices with relatively short pulse durations, the spin–orbital angular momentum coupling effects are expected to be weak. In general, such spin–orbital angular momentum coupling effects would occur significantly by the tight focusing of optical vortices with a relatively long pulse duration^43^. Further discussion of spin–orbital angular momentum coupling effects in monocrystalline silicon needle formation will require additional experiments being performed by employing highly focused submicrosecond optical vortex pulses.

## Conclusion

To the best of our knowledge, this study is the first to successfully demonstrate the formation of a monocrystalline silicon needle (height, ~14 μm; thickness, ~2.9 μm; tip curvature, ~200 nm) recrystallized by irradiation with a picosecond vortex pulse (single vortex pulse deposition). We also revealed for the first time how the silicon needle is formed. The picosecond vortex pulse deposition forces the melted silicon to be directed toward the core and establishes the needle on a microsecond scale. The height of the needle was also enhanced by superimposing several vortex pulses on the target, reaching approximately 40 μm.

Such a monocrystalline silicon needle has never been fabricated by conventional Gaussian mode illumination, and it can be applied to devices in many fields, such as core–shell devices for silicon photonics and photovoltaic devices as well as nano- or microelectromechanical systems.

## Methods

**Figure 7:**
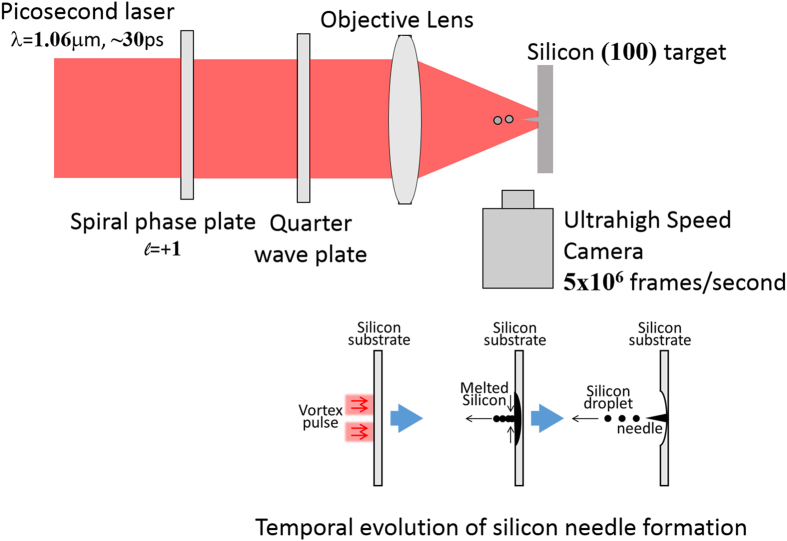
Experimental setup for silicon needle fabrication.

Figure 7 shows an experimental setup for silicon needle fabrication. The pump laser used in this study was also a conventional Q-switched mode-locked Nd:YAG laser (B.M. industries, Series 5000) with a wavelength of 1064 nm, a pulse repetition rate of 10 Hz, and a pulse duration of 20 ps. Its output was converted to a right-handed optical vortex with a topological charge, *l*, in the range of +1 by using a spiral phase plate (RPC photonics, VPR-m1064) that provided an azimuthal phase of 2π. A quarter-wave plate also enabled suppression of the polarization dependence of the ablation efficiency as well as constructive addition of the spin angular momentum associated with the circular polarization to the optical vortex pulse. The measured output energy on the target surface was 0.1–1.6 mJ. The circularly polarized optical vortex pulse was focused as a spot with a diameter of approximately 60 μm on the Si target by an object lens with a numerical aperture of 0.13.

The target used was a polished (100) monocrystalline silicon plate with dimensions of 24 mm × 12 mm × 0.67 mm. The ablated surface of the Si plate was coated with platinum by a sputtering device (JEOL, JFC-1600 Auto Fine Coater) and observed with a scanning electron microscope (JEOL, JSM-6010LA) with a spatial resolution of 8 nm at 3 kV. All experiments were performed at atmospheric pressure and room temperature. The temporal dynamics of the silicon needle formation was visualized using an ultrahigh speed camera, operating at a rate of 5 × 10^6^ frames/s (Shimadzu Corp., Hyper Vision HPV-X).

## Additional Information

**How to cite this article**: Takahashi, F. *et al.* Picosecond optical vortex pulse illumination forms a monocrystalline silicon needle. *Sci. Rep.* **6**, 21738; doi: 10.1038/srep21738 (2016).
